# Grasping learning, optimization, and knowledge transfer in the robotics field

**DOI:** 10.1038/s41598-022-08276-z

**Published:** 2022-03-16

**Authors:** Luca Pozzi, Marta Gandolla, Filippo Pura, Marco Maccarini, Alessandra Pedrocchi, Francesco Braghin, Dario Piga, Loris Roveda

**Affiliations:** 1 Bioengineering Department, Politecnico di Milano, 20133 Milano, Italy; 2 Mechanical Department, Politecnico di Milano, 20133 Milano, Italy; 3 Istituto Dalle Molle di studi sull’Intelligenza Artificiale (IDSIA), Scuola Universitaria Professionale della Svizzera Italiana (SUPSI), Università della Svizzera Italiana (USI), 6962 Lugano, Switzerland

**Keywords:** Mechanical engineering, Engineering, Electrical and electronic engineering

## Abstract

Service robotics is a fast-developing sector, requiring embedded intelligence into robotic platforms to interact with the humans and the surrounding environment. One of the main challenges in the field is robust and versatile manipulation in everyday life activities. An appealing opportunity is to exploit compliant end-effectors to address the manipulation of deformable objects. However, the intrinsic compliance of such grippers results in increased difficulties in grasping control. Within the described context, this work addresses the problem of optimizing the grasping of deformable objects making use of a compliant, under-actuated, sensorless robotic hand. The main aim of the paper is, therefore, finding the best position and joint configuration for the mentioned robotic hand to grasp an unforeseen deformable object based on collected RGB image and partial point cloud. Due to the complex grasping dynamics, learning-from-simulations approaches (e.g., Reinforcement Learning) are not effective in the faced context. Thus, trial-and-error-based methodologies have to be exploited. In order to save resources, a samples-efficient approach has to be employed. Indeed, a Bayesian approach to address the optimization of the grasping strategy is proposed, enhancing it with transfer learning capabilities to exploit the acquired knowledge to grasp (partially) new objects. A PAL Robotics TIAGo (a mobile manipulator with a 7-degrees-of-freedom arm and an anthropomorphic underactuated compliant hand) has been used as a test platform, executing a pouring task while manipulating plastic (i.e., deformable) bottles. The sampling efficiency of the data-driven learning is shown, compared to an evenly spaced grid sampling of the input space. In addition, the generalization capability of the optimized model is tested (exploiting transfer learning) on a set of plastic bottles and other liquid containers, achieving a success rate of the 88%.

## Introduction

Recent advancements in robotics have pushed this field beyond the traditional paradigm of industrial robots, repeating a predefined task in a known and controlled environment. The development of robust artificial intelligence algorithms allows endowing robots with generalization capabilities, an essential skill for robots meant to operate semi- or fully autonomously in conditions that are not known a priori. As they are meant to assist people in their everyday activities, service robots perfectly match this description, having to act in an unpredictable environment to accomplish useful tasks, possibly involving human-robot interaction.

The introduction of affordable components led to the spread of domestic service robots and enabled them to reach the homes of more and more people, where robots are helpful in housekeeping, cooking, and acting as companions^1^. The phenomenon likewise concerns public places such as airports and hotels, that have recently witnessed the deployment of robots as guides for travelers^2^. A further sector that benefits from the advancement in robotics is the healthcare and assistance sector. Robotics could be an effective mean to face the ageing of the population, helping frail people, especially the elderly, to maintain an independent life at their own place. The robots can also support the clinicians in healthcare facilities to help in inpatient monitoring, entertainment, logistics and cleaning, relieving the workload of nurses and allowing them to focus on the patients’ care^3,4^.

Given the large number of everyday activities that require interaction with the environment, service robots without manipulation capabilities are limited in use and independence. Object manipulation, however, is not an easy requirement to satisfy. Though investigated for decades^5,6^, robotic manipulation is still an open issue in the scientific community. Moreover, the wide variety of tasks involving manipulation and the multitude of objects with which we interact in everyday life make it necessary to enable the robot to work in conditions that are, at least partially, unknown. To address this complex problem, several types of compliant grippers have been developed^7,8^. These devices promise to greatly simplify the problem of grasping, as they can adapt to the environment, avoid damages tp breakable objects, and allow to grasp deformable ones. In contrast to rigid ones, the joint configuration of a soft gripper cannot be fully determined, thus alternative control strategies must be introduced. For anthropomorphic end-effectors, these control strategies are often inspired to the human behavior (*i.e.*they exploit human synergies) and are embedded into the gripper mechanical design^9^. This kind of end-effectors can achieve very good results when used by a human operator, while autonomous grasping still represent a challenging research topic. A promising solution is to leverage on machine learning techniques to endow robots with self-adaptation capabilities. In this view, the possibility to adapt the manipulator behavior on the fly, when an unforeseen problem is encountered, has been investigated in recent years.

Given the depicted situation, the presented work proposes a vision-based framework for grasp learning of deformable objects. To prove the validity of the method, the obtained grasps are tested on a service robot to accomplish a relevant task.

## Related work and paper contribution

Learning-based manipulation is an extensively covered topic in the scientific literature. In contrast to the traditional model-based approaches that rely on a rich prior knowledge of the object, data-driven approaches aim to gain this knowledge from data.

The combination of mechanical adaptation and machine learning techniques offers an intriguing solution to the problem of robotic grasping. In this framework, a multi-fingered compliant end-effectors had been used jointly with a convolutional neural network predicting the optimal gripper pose from objects’ point clouds, reaching an 87% success rate on unseen objects^10^. Moreover, soft grippers have shown impressive adaptation capabilities when trained on a dataset of grasping primitives, either acquired by manually operating the gripper^11^ or from video recordings of human grasps. However, these approaches often rely on sensors (e.g. inertial measurement units) to detect the contact with the object^11,^. Most importantly, these human-inspired methods strongly depend on the mechanical design of the end-effector. Indeed, the possibility to apply the same grasp type to considerably different objects is heavily influenced by the reliability of the “*intelligence embodied in the hand mechanics*”^12^.

Algorithms from the family of reinforcement learning (RL) methods have been proven effective in various manipulation tasks. In RL algorithms, an agent (i.e. the whole robot, the manipulator or its end-effector) goes through a trial-and-error process to identify a behavioral policy. The policy determines the next action of the agent, given the current state of the environment and of the agent itself, in order to maximize a reward. The combination of RL and deep convolutional neural networks gave birth to deep reinforcement learning (DRL) methods that can learn a policy from large inputs, thus enabling to address more complex problems. Starting from vision data, DRL has been employed to learn an end-to-end pickup task on a mobile manipulator^13^ and to achieve the closed-loop control of a grasping task^14^. DRL algorithms have been also exploited to learn dexterous manipulation tasks^15^ as well as to learn policies both to select the grasping strategy for the object at hand and to control its execution^16^. The main issue that arises when working with DRL is the huge amount of samples required for the training. Collecting these samples on several robots working in parallel, as in^14^, is oftentimes a non-viable solution. Thus, a widely adopted strategy is to train the robot in simulation^16^, but transferring the learned policy to the real robot can result in a considerable drop in performance^13^. Another possibility is to initialize the policy with human demonstration to reduce the training^15,16^. Though effective, this shrewdness is hard to adapt to compliant end-effectors in which the joint configuration cannot be completely controlled.

The DRL can also take advantage of haptic exploration to learn a grasping strategy. The scientific literature on this topic shares the idea to exploit tactile data to achieve the closed-loop control of the grasp. Starting from this common ground, diverse methods have been proposed, as the learning can lean only on feeling the contacts^17^, or start from a coarse vision-based positioning of the end-effector^18^, and it maybe carried out in simulation^19^ or on a real robot^18^. The clear drawback of these appealing methods is the need to have a sensorized end-effector, resulting in an increase of the hardware costs.

Besides RL, other optimization algorithms have been used to address the problem of robot manipulation, including Bayesian Optimization (BO). Regarding the BO applications in robotic manipulation, the auto-tuning of the control parameters for an industrial manipulator is proposed by Roveda and colleagues^20^, Drieß et al. employed BO to tune force controllers for combined position/interaction tasks^21^, and Petit and co-workers^22^ leveraged BO to grasp from a cluttered bulk composed of the same object instance using a robotic arm and a parallel gripper. Aside from industrial applications, a Bayesian approach has been adopted in several works to determine the optimal grasp point for lightweight robots. Kroemer and colleagues^23^ combined a BO and imitation learning to implement an hybrid system made of two controllers. The high-level controller selects the grasp location, while the low-level controller is responsible for the execution of the grasp and leverages on imitation learning from human demonstrations. Starting from planar visual information, Montesano and co-workers^24^ developed a method for the active learning of the grasp point, testing its effectiveness on a humanoid robot. Nogueira et al.^25^ studied the use of BO to position an iCub hand in order to safely grasp an object. The proposed method is evaluated in simulation and does not rely on visual information. The optimization returns the 2D translation to bring the hand in the best position to perform a pre-defined power grasp. Said procedure demonstrates its effectiveness on several modeled objects, and it has been extended to the 3D case by a subsequent study by Castanheira et al.^26^.

In the framework of the current literature, the presented work is centered on the vision-based manipulation of non-rigid (*i.e.*, deformable) objects with a multi-fingered, underactuated, compliant, sensorless (*i.e.*, no force/torque measurements are available) hand. The goal is to train a robust and task-oriented grasping strategy, and to leverage on transfer learning (TL) to apply the acquired knowledge on objects that are unknown to the model.

The main contribution of the present paper are

- A BO-based grasp learning method
  (i) Optimizing the joint configuration of a compliant, underactuated end-effector;
  (ii) Working with deformable and (partially) unknown objects;
  (iii) Not relying on simulated trials;
  (iv) Requiring minimal sensory information.
- An application for said grasp learning method on a service robot.

The authors propose an optimized learning approach, which allows to carry out the training directly on the robot (*iii*). In fact, due to the complex interaction dynamics involving the compliant gripper and the deformable object, simulations-based training algorithms (*e.g.*, RL) are not effective for the given task. BO is deemed the ideal candidate algorithm thanks to its sample efficiency (*i.e.*, minimizing the experimental trials) as DRL approaches would require an unbearable training time if not properly initialized. Such initialization can come neither from simulation, given the cumbersome work needed to simulate both the passive component of the hand behavior and the deformability of the objects (*i*)(*ii*), nor from imitation or kinesthetic learning, as the compliant hand could end up in very different configuration even if the same set of joint values is provided (*i*). The presented solution is designed to work with a minimal amount of real-world data and a coarse (but meaningful) initialization of the desired grasping point. The hand does not lean on tactile sensors, indeed the grasping task is entirely driven by visual data acquired with a RGBD camera and does not require any previous knowledge of the object model (*iv*).

Though the potentiality of a Bayesian approach to the optimization of grasping tasks has been long since foreseen^27^, it has been seldom exploited as a stand-alone learning algorithm. To the best of the authors’ knowledge, the most relevant examples of grasp strategy optimization on an anthropomorphic end-effector using BO are represented by the works from Nogueira et al.^25^ and its prosecution and extension by Castanheira et al.^26^, previously described. As in other important contributions regarding BO and robotic grasping^23,24^, the focus of these works is the determination of the end-effector positioning, and not the optimization of the joint configuration of the gripper (i). To date, proposed solutions to the latter problem involve kinematic synergies^25,26^, not available for the problem at hand.

## Problem description

The final goal of this work is to endow TIAGo with the grasping capability needed to pour water from a plastic bottle. The reason why this task has been selected is twofold: *(i*) it requires the functional manipulation of a non-rigid object; (*ii*) it is a significant task for a robot providing assistance to frail people. To further clarify the statement (*i*), the non-rigid behavior is the one shown by a common plastic bottle when squeezed. The *functional manipulation* of the bottle refers to the sequence of actions that are needed to actually pour the liquid in a glass/cup. Identifying a grasping strategy that satisfies the requirements is not trivial as the straightforward choice of a very tight grasp results in the excessive squeezing of the bottle. This results in turn in the bottle slipping in the hand during the grasp and may lead to a deformation that would compromise the object’s functionality. On the other hand, not providing enough grasping force would not provide enough stability to fulfill the task.

**Figure 1:**
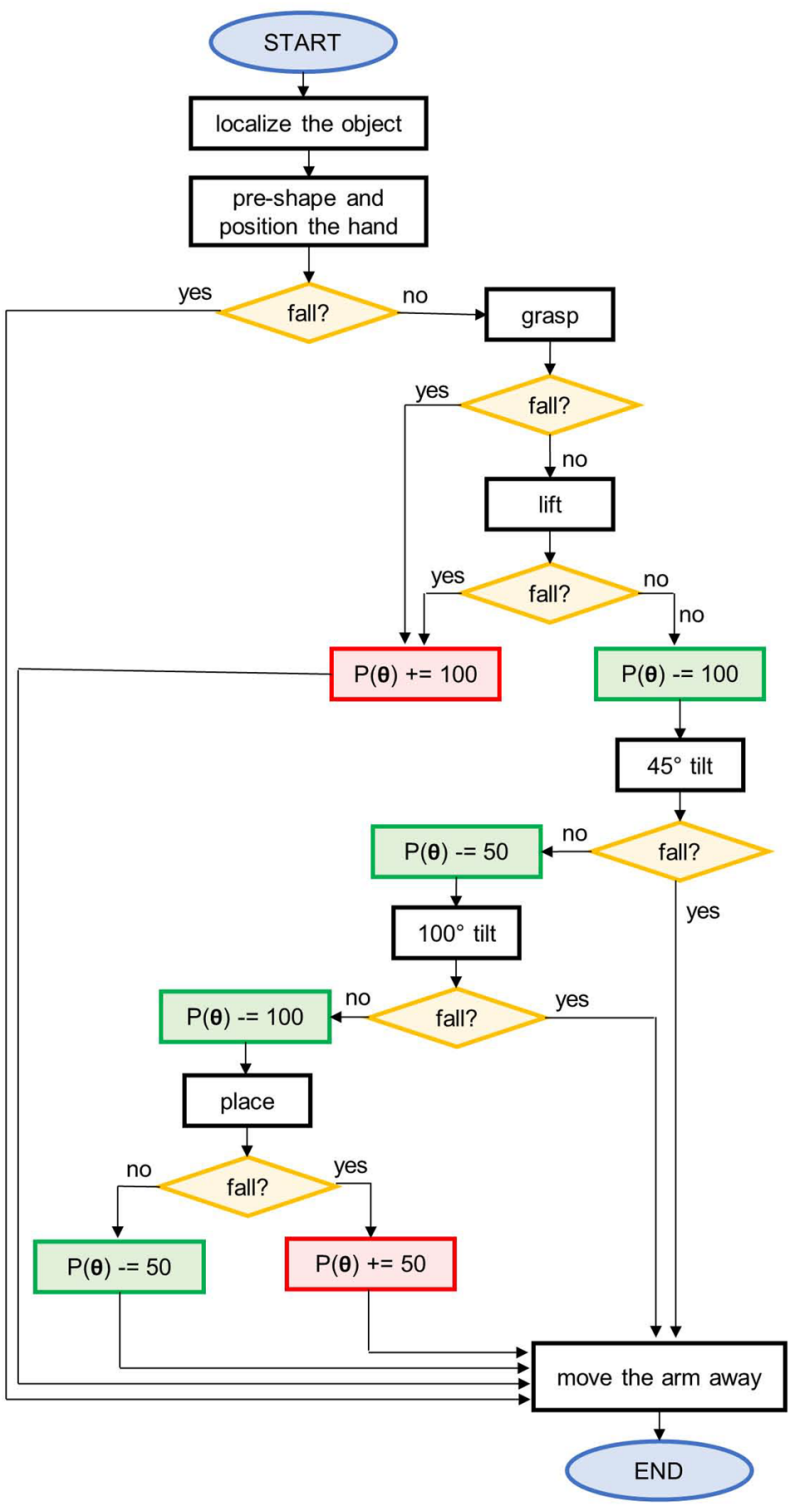
Block diagram of the task, here detailed step by step: (i) *localize the object*, the object’s position, radius and height are assessed from RGBD data; (ii) *pre-shape and position the hand*, the thumb is opposed and the hand placed in the desired grasp point. (iii) *grasp*, the joints of the hand are moved according to the parameters in ${\varvec{\theta }}$; (iv) *lift*, the hand is raised; (v) 45° tilt, the arm is brought to a pre-defined configuration, then the wrist is rotated by 45°; (vi) 100° *tilt*, the wrist is rotated to achieve a 100° of the bottle; (vii) *place*, the arm returns to the configuration in (iv), the hand then moves down and opens to release the object; (viii) *move the arm away*, the hand is distanced from the object.

To evaluate the performance of each set of parameters ${\varvec{\theta }}$, defining a grasping strategy, a discrete penalty function $P$ is defined. The optimization process has been designed as a minimization process, hence the values of $P$ are distributed in a *the-lower-the-better* fashion, with negative values conceptually representing rewards. The structure of the evaluation procedure for a set of parameters ${\varvec{\theta }}$ is schematized in Fig. 1. As can be inferred from Fig. 1, failures in the early stages are penalized with a $P({\varvec{\theta }}) = +100$, while reward values depend on the most advanced successful stage of the task (*i.e.*, lift − 100; 45 tilt − 150; 100 tilt − 200; place − 300). The uneven steps in the penalty function have been conceived so to drive away the algorithm from the worst strategies and avoiding it to settle for suboptimal solutions.

## Methodology

### Bayesian optimization

A *grasping strategy* is defined by a set of the parameters ${\varvec{\theta }}$ and its performance can be evaluated to obtain the value of $P({\varvec{\theta }})$. The search for the optimal grasping strategy then reduces to the minimization of the penalty function $P({\varvec{\theta }})$ with respect to ${\varvec{\theta }}$, within a space of admissible values ${\varvec{\Theta }}$, hence

1 $$\begin{aligned} {\text {arg}}\underset{{\varvec{\theta }}\in {\varvec{\Theta }}}{{\text {min}}}\,P({\varvec{\theta }}) \end{aligned}$$

However, an expression of the cost $P$ as a function of the parameters vector ${\varvec{\theta }}$ is not available in closed-form. Furthermore, this cost cannot be evaluated through simulations as the dynamics of the passive behavior of the end-effector are hard to model with a sufficient level of accuracy. Instead, it is possible to perform experiments on the robot to evaluate the penalty $P_i$ achieved for a given parameters vector ${\varvec{\theta }}_i$, and thus exploit the measurements of $P$ to drive an optimization algorithm. The chosen optimization algorithm must fit the characteristics of the problem at hand, namely:

(i): an analytical expression of the penalty $P$ as a function of the tuning parameters ${\varvec{\theta }}$ is not available;
(ii): there is no evidence that the function $P({\varvec{\theta }})$ has any particular structure, as linearity or convexity;
(iii): the function is expensive to evaluate.

Features (*i*) and (*ii*) preclude the employment of classical gradient-based algorithms and lead to resort to a gradient-free and data-driven global optimization algorithm for black-box functions. Within this class of algorithms, *Bayesian optimization* (BO) is known to be the most efficient in terms of the required number of function evaluations^28^. The sample efficiency of BO makes it the most promising approach to deal with (*iii*). In this work, the function is said “expensive to evaluate” referring to the human operator’s time, though the same considerations apply to experiments with a high monetary or computational cost.

In BO, the cost $P$ is simultaneously learned and optimized by sequentially performing experiments on the robot. Specifically, at each iteration *i* of the algorithm, an experiment is performed for a given grasping strategy ${\varvec{\theta }}_i$ and the corresponding cost $P_i$ is measured. All the collected parameters-penalty pairs build up a dataset of observations ${\mathcal {D}}_i = \{({\varvec{\theta }}_{1}, P_{1}), ({\varvec{\theta }}_{2}, P_{2}), \dots , ({\varvec{\theta }}_{i}, P_{i})\}$. A *surrogate* statistical model, here a Gaussian Process (GP) model, is fitted to the data to describe the relationship between the input parameters ${\varvec{\theta }}$ and the penalty $P({\varvec{\theta }})$, *i.e.*,

2 $$\begin{aligned} P({\varvec{\theta }}) \sim GP({\bar{P}}({\varvec{\theta }}), \kappa ({\varvec{\theta }}',{\varvec{\theta }})), \end{aligned}$$

where ${\bar{P}}({\varvec{\theta }})$ and $\kappa ({\varvec{\theta }}',{\varvec{\theta }})$ represent the estimated mean and the covariance functions of the GP, respectively. The surrogate GP model is then used to select the subsequent point to sample ${\varvec{\theta }}_{i+1}$ by maximizing the *expected improvement* (EI). EI allows to deal with the exploration-exploitation trade-off, balancing the will to learn more about the value of $P$ in the regions of the input space with high variance, and the intention to select an input that possibly minimizes the penalty.

### Transfer learning

To reduce the training time when a novel object is encountered, a GP model is fitted on the previously collected data and used as a prior to estimate the new surrogate GP.

For this purpose, the difference between the measured cost $P_i$ and the mean of the previously estimated GP model ${\overline{P}}^{\mathrm{old}}({\varvec{\theta }}_i)$ is computed, *i.e.*,

3 $$\begin{aligned} \Delta P_i = P_i - {\overline{P}}^{\mathrm{old}}({\varvec{\theta }}_i). \end{aligned}$$

In a new optimization phase, the Gaussian Process describing the cost variation $\Delta P({\varvec{\theta }})$ is estimated as

4 $$\begin{aligned} \Delta P({\varvec{\theta }}) \sim GP(\Delta {\overline{P}}({\varvec{\theta }}), \kappa _{\Delta }({\varvec{\theta }}',{\varvec{\theta }})), \end{aligned}$$

where $\Delta {\overline{P}}({\varvec{\theta }})$ and $\kappa _{\Delta }({\varvec{\theta }}',{\varvec{\theta }})$ are the mean and covariance function of the GP describing the cost variation $\Delta P({\varvec{\theta }})$.

Finally, the updated surrogate model of the cost $P$ can be expressed as

5 $$\begin{aligned} P({\varvec{\theta }}) \sim GP({\overline{P}}^{\mathrm{old}}({\varvec{\theta }}) + \Delta {\overline{P}}({\varvec{\theta }}), \kappa _{\Delta }({\varvec{\theta }}',{\varvec{\theta }})), \end{aligned}$$

This GP model of $P({\varvec{\theta }})$ is eventually used by the Bayesian optimization algorithm to find the next point to sample ${\varvec{\theta }}_{i+1}$.

#### Remark 1

Note that estimating the cost variation $\Delta P({\varvec{\theta }})$ and then adding ${\overline{P}}^{old}({\varvec{\theta }})$ is equivalent (up to the choice of the hyperparameters defining the kernel matrix $\kappa _{\Delta }$) to train a GP model with a prior mean ${\overline{P}}^{old}({\varvec{\theta }})$.

## Experimental evaluation

### Experimental setup

**Figure 2:**
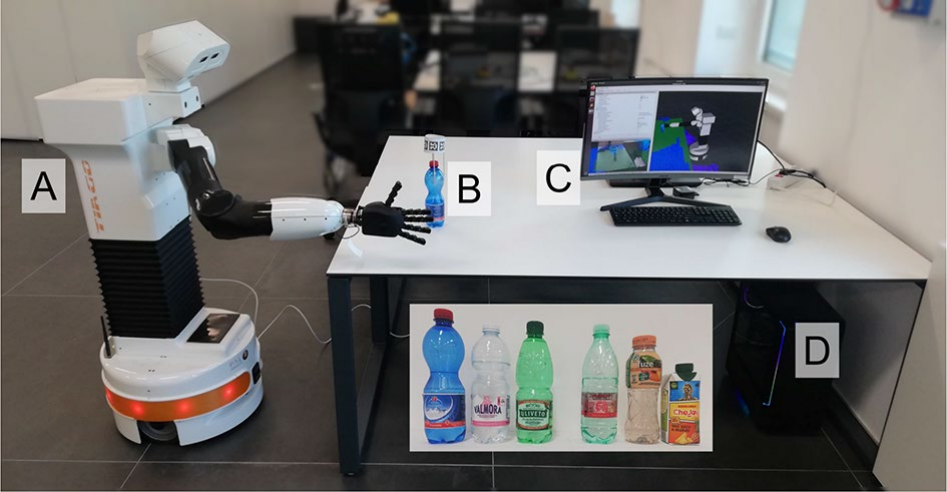
The experimental setup used to train the model. (**A**) The TIAGo mobile manipulator. Its 7 DoF arm is used to position the Hey-5 hand above the table plane and close to the object to grasp. (**B**) The bottle to grasp and the ring of ArUco markers enabling the automatic detection of the task outcome. (**C**) The monitor of the workstation, displaying the MoveIt planning scene, i.e. the environment as seen by the robot, used for the arm motion planning and collision avoidance. (**D**) The workstation, connected to TIAGo via Ethernet. In the box in the bottom, the bottles used to test the method are shown. From left to right, the objects are named *bottle A*, *B*, *C*, *D*, *E* and *juice box*.

The TIAGo robot is a mobile manipulator produced by PAL Robotics (see Fig. 2)^29^. For the purposes of the study, only a part of its sensory and motion capabilities is utilized. The manipulator is made of a 7 degrees of freedom (DoF) arm, mounting an anthropomorphic, underactuated, compliant hand called Hey-5 hand (developed by PAL Robotics with contributions from QRobotics, derivated from the Pisa/IIT SoftHand open source project^30^). The Hey-5 hand has a total of 19 DoF, only 3 of which are actuated: one for the thumb, one for the index, and one for the middle, the ring and the little together. The remaining 16 DoF are driven by elastic bands acting as tendons, making the hand compliant and able to self-adapt to the shape of the objects. The vision data that drives the task comes from the RGBD camera (Orbecc Astra S) integrated into the head of TIAGo.

Just during the training procedure, a ring of ArUco markers is attached to the bottle. The recognition of said markers allows automatizing the detection of the task’s outcome, as the robot is able to autonomously identify failures by sensing the marker positions. In this way, the penalty function is automatically evaluated during training, and the user intervention is reduced to the repositioning of the bottle in case of falls.

The described setup is illustrated in Fig. 2.

### Optimized parameters

**Table 1:** The range of values and discretization step for each optimized parameters.

Parameter	Unit	Range	Optimization range	Step
Thumb joint	rad	0.0–6.1	1.6–6.1	0.1
Index joint	rad	0.0–6.7	1.6–6.7	0.1
Middle, ring, little joint	rad	0.0–9.1	1.6–9.1	0.1
Grasp point height	cm	–	− 1.5 ± 1.5	0.5

The optimization process involves 4 parameters:

(i): the target joint value to be sent to the actuator of the thumb;
(ii): the target joint value to be sent to the actuator of the index;
(iii): the joint value to be send to the unique actuator moving the middle, ring and little fingers at once;
(iv): the height of the grasp point in the range of the height at which the bottle body has the smaller radius.

The range of (*i*), (*ii*) and (*iii*) is manually narrowed to exclude the configurations in which the closure of the fingers would be far too little. The central value for (*iv*) is measured manually on the bottle and the explored region spans from 1.5*cm* below to 1.5 cm above said point. The resulting ranges of the optimized parameters, together with the discretization steps, are shown in Table 1.

#### Remark 2

The *manual narrowing* excludes those configurations that, based on everyday experience, would for sure led to a failure (e.g. when the thumb is not in opposition). However, avoiding to exploit this prior knowledge would have a minimal impact as the algorithm would soon stop to explore the concerned region.

#### Remark 3

Though the optimized variables are intrinsically continuous, the input domain is discretized taking into account the hardware characteristics. The limited accuracy of sensors and actuators sets a lower bound for the discretization step. Any further decrease, would not go together with an improvement in the success rate of the target task execution.

The iterative optimization of the depicted parameters is carried out from scratch on different bottles. Then, the individual models are combined into a single global model in which the radii of the bottles at the grasp point have been included as a further additional parameter. Therefore we have an additional parameter:

(v): the radius of the bottle at the tighter point of the object’s body.

This last parameter does not actually undergo any optimization (that would be meaningless, as it depends from (*iv*)). Instead, it is exploited to do predictions: given the radius of a new bottle at the grasp point, the model returns the optimal grasping strategy.

### Experimental protocol

Three training sessions have been carried out without any prior information on three different plastic bottles (visible in the box in Fig. 2). Each training session starts with an initialization phase, consisting of the evaluation of 5 grasping strategies drawn at random from the input space, followed by a two-stage training. The first stage is composed of 50 iterations, each one evaluating a single set of parameters. The model returned by the first stage is used to predict the expected penalty for each possible strategy ${\varvec{\theta }}$ in the search space ${\varvec{\Theta }}$. The domain for the second stage of the optimization is shrunk to the smaller hyperectangle including all the strategies with a predicted penalty $P({\varvec{\theta }})\le -200$ (i.e. believed to lead, at least, to a successful tilt). With the resulting restricted domain, further 25 iterations are carried out.

By the end of the training, 80 samples are collected (5 for the initialization, 50 in the first stage and 25 in the second stage). Each sample is made of the optimized parameters (*i*), (*ii*), (*iii*), (*iv*) (as described in “Optimized Parameters”) and the associated penalty value. To each sample, the parameter (v) (see “Optimized Parameters”) is added. After doing this for all the three independently learned models (hereinafter *individual models* or *object-specific models*), a dataset of 240 samples has been obtained. A new GP is fitted on said data (hereinafter *global model*) in the attempt to include in a single model all the information learned from the three objects, and it is therefore used to predict the optimal grasp strategy for a novel object, given the radius at the grasp point. The possibility to fine-tune the global model (with the algorithm in Transfer Learning) on a specific bottle through 20 additional iterations is explored for a single bottle.

A Matérn kernel is used in the GP models describing the surrogate of the penalty function ${\overline{P}}^{old}({\varvec{\theta }})$ and its variation $\Delta P({\varvec{\theta }})$, with kernel hyperparameters computed by maximing the marginal likelihood^31^[Ch. 5.2]. These hyperparameters are updated at each iteration of the BO algorithm.

### ROS implementation

The presented approach has been developed by implementing ROS nodes in Python language to manage the vision system, the manipulator motion and the BO process (as described in “Bayesian Optimization”).

The vision system leverages the *Detectron2* library from Facebook Artificial Intelligence Research^32^, employing a Mask R-CNN for instance segmentation. Sending the RGB images from the camera stream through the neural network returns the mask of the object to grasp. The mask is applied to the depth image to retrieve the object position, maximum radius and height, under the assumption that the object can be modeled as a rotational symmetry.

The movements of the arm and the end-effector are controlled using *MoveIt!*^33^ for motion planning with collision checking. The information about the object’s position and geometry is used to update the planning scene, allowing the robot to come in contact with the object.

### Validation

To evaluate the performance of both the individual models and the global model, the optimal grasp strategy predicted by each model has been tested with the following procedure. For the validation test, the grasping strategy is tested with the object placed in 5 different positions, with the central one lying in front of the robot and the others shifted by 10*cm* along two directions on the table plane. In each position the test is repeated 5 times, for a total of 25 repetitions of the task. Moreover, a grid search collecting 375 evenly spaced samples over the whole domain is conducted on a single bottle, to compare its performance in terms of the required number of iterations, grasp quality and generalization capability with the proposed approach. During the validation tests, two differences with respect to the training must be underlined: (*i*) there are no markers attached to the bottle; (*ii*) before putting the bottle down on the table, during the repositioning phase, the grasp of the middle-ring-little joint is partially released. The latter is a trick to help the robot during the most failure-prone phase of the task: the middle-ring-little joint is the main culprit of the bottle in-hand inclination, as the thumb and the index actions balance each other out. Nevertheless, this operation is not included in the learning to let the robot identify strategies that prove capable of carrying out the whole task without any aid.

## Results

In this section the quantitative evaluation of the performance of the optimization algorithm tested on the TIAGo robot is presented both in the training and validation procedure. A video is available at https://www.youtube.com/watch?v=SfT2lyhFuuQ.

**Figure 3:**
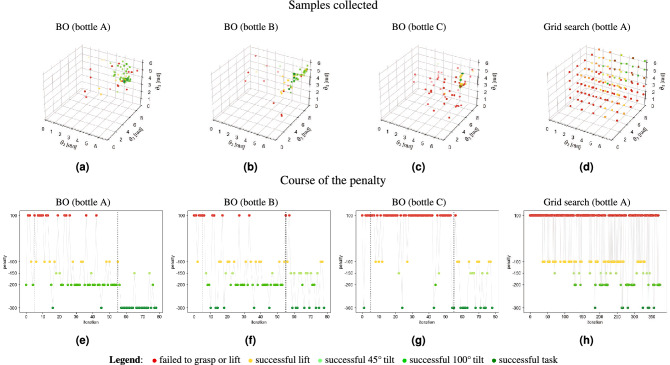
The plots in the top line depict the distribution of the collected samples over the domain during a run of the corresponding optimization method. In (**a**), (**b**) and (**c**) the grasping strategies and the corresponding evaluations collected in the training of the individual models are shown, whereas in (**d**) the result of the evenly spaced exploration of the domain is reported. In the bottom line, the course of the penalty during the training procedure are shown: (**e**), (**f**), (**g**) report the results of the BO runs, and (**h**) the outcome of the grid search. The vertical dashed lines in (**e**), (**f**) and (**g**) demarcate the phases of the training (in order: initialization, first and second stage).

Figure 3 provides a visualization of the distribution of successful/unsuccessful strategies within the domain, and shows the course of the penalty function during the training of each one of the individual models and the grid search, conducted using bottle A as the object to grasp.

**Figure 4:**
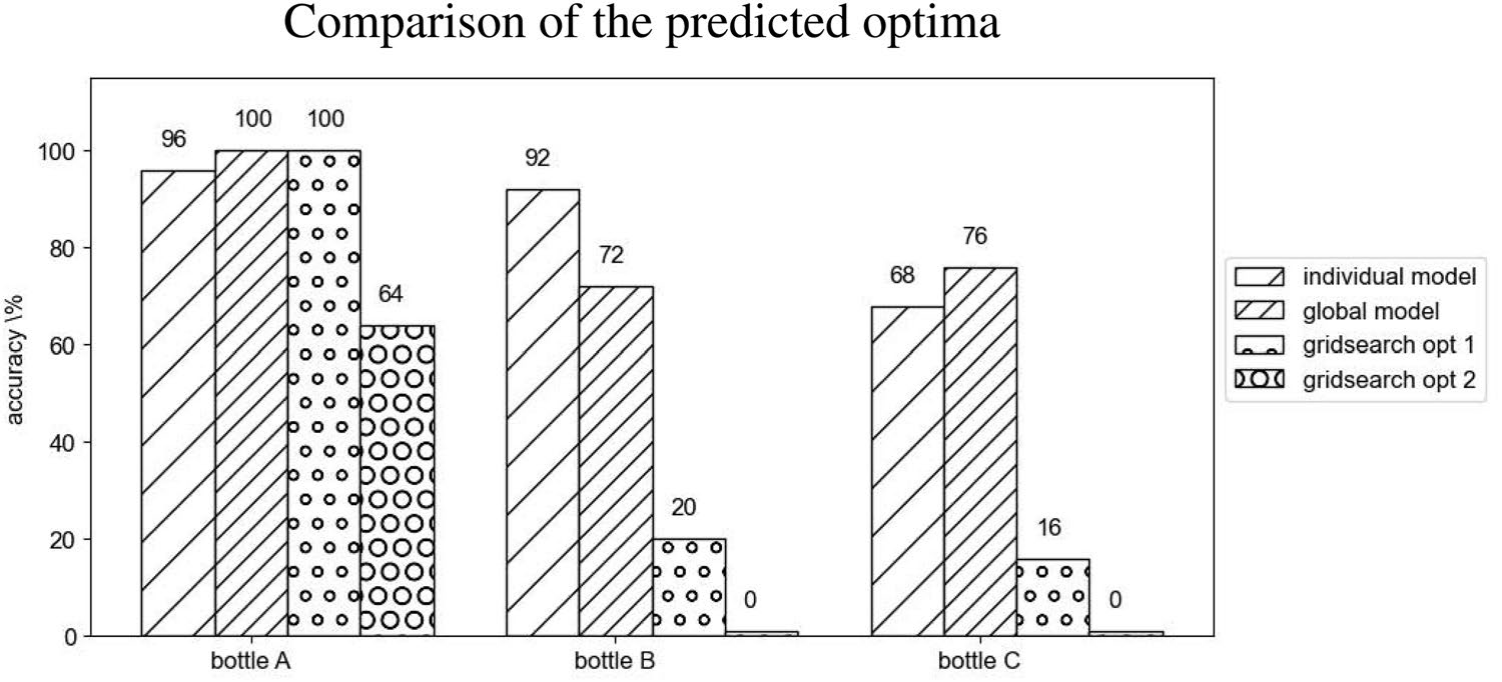
Accuracy of the optima predicted by each method.

In Fig. 4 the accuracies in the validation test shown by each model are compared. To evaluate the grid search, lacking a univocal definition of optimum, 2 random configurations are taken from the 9 successful ones found in the search.

**Figure 5:**
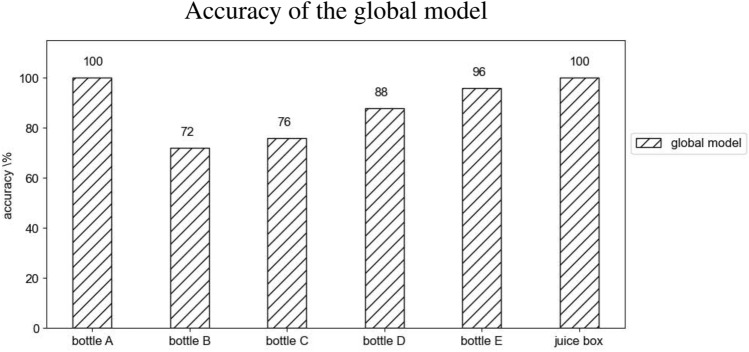
Accuracy of the optima predicted by the global model on each object. The global model is obtained merging the observations made during the training of the individual models for bottles A, B and C. The remaining objects are completely new to the model.

In Fig. 5 the accuracy of the optima predicted by the global model for each one of the tested objects is presented. Overall, the model is able to provide a successful strategy in the 88% of the cases. The additional training of the global model to fine-tune it on a specific object is carried out on bottle D. The result after 20 further iterations is an accuracy of the 76% (compared to the 88% obtained previously). The results in Fig. 5 for bottle D are complemented by a specification: the object is likely to escape from the hand when the grasp is loosened before the repositioning. Despite that, as the fingers are still surrounding the object, they can be effective to prevent the fall, hence the bottle ultimately leans against the table upright and the task is considered to be successfully accomplished.

## Discussion

The results outlined in Results demonstrate the efficiency of the proposed method in terms of the number of samples and hence required time for computation. The part of Fig. 4 relative to bottle A (on which the grid search has been carried out), emphasizes how the learned model is able to obtain comparable (if not better) results with a much lower number of evaluations compared to the grid search (respectively 80 and 375 samples). During the BO run on bottle A, the 26.25% of the iterations resulted in successful tasks, a rate that dramatically drops to the 2.4% in the grid search. Moreover, the discretization steps of the grid search are up to 15 times larger than the ones of BO (reported in Table 1), in order to explore the whole domain uniformly in an achievable number of iterations. This gap in granularity can be closed only at the cost of a tremendous increase in the number of samples to collect that would ultimately make the search unfeasible in practice.

Focusing on the quality of the obtained solution, it should be noticed how one of the two optima resulting from the grid search is significantly worse than the other strategies for bottle A (a drop of the 32–36% in accuracy is observed). This observation draws the attention to another important benefit of the presented solution: though continuing to explore even during the advanced stages of the training, the BO algorithm focuses the sampling on the most promising region of the input space. Together with the shrinkage of the domain, this allows testing very similar (or even identical) strategies more than once, hence discriminating the most robust ones.

The effectiveness of the selected two-stage training strategy can be appreciated in Fig. 3e–g, observing the consistent improvement of the penalty function in the passage from the first to the second phase (represented by the rightmost dashed line in each plot). The course of the penalty seems to follow the same pattern in the grid search as well, but this is due to the fact that the exploration begins in the bottom left corner of Fig. 3d and ends in the top right one, where the best strategies accidentally are found. The same reasoning does not apply to the course of the reward guided by BO, as the starting point of the process is drew at random and the exploration does not follow a pre-defined path.

As far as the generalization capability is concerned, the global model demonstrates to be applicable to novel objects, though provided with minimal information and a few assumptions on the object shape. Conversely, Fig. 4 allow appreciating how, rather predictably, the outcome of the grid search results in disastrous performance when applied on unforeseen, though similar, objects. The comparison of the performance of the object-specific models and the global model shows an almost unchanged accuracy for bottle A and bottle C. Differently, an accuracy drop of the 20% is measured for bottle B. From this observation arises the need to balance the trade-off between the generalization capabilities of the solution and the accuracy on a single object. To face this issue, the model has been used as a starting point to fine-tune an object-specific strategy for one of the novel objects (bottle D). Though the presented preliminary result is not satisfactory (the accuracy is actually slightly worsened), the authors consider the object-specific refinement of the strategy as a promising solution that deserves a deeper investigation before being discarded.

The accuracies summarized in Fig. 5 evince the applicability of the presented method to all the considered objects, including the ones (bottle D and juice box) that are not shaped to facilitate the grasp or are made of a different material (juice box).

The discussed quantitative results are complemented with qualitative observations made during the training and validation phases about: (*i*) the hand positioning; (*ii*) the grasp relaxation during the repositioning phase. As regards (*(i)*), the positioning of the hand heavily affects the outcome of the task for the bottles that do not have a waisted profile, in which the facilitated grasp point is represented by a narrow groove (bottle B and C). For this kind of objects, the repeatability error of the manipulator (3.5 mm in the worst-case scenario, as reported by the producer) may lead to the end-effector misplacement, causing the index finger not to fall into said groove. As a consequence, the bottle is not held firmly and the task fails during the lift or the manipulation. Looking at (*(ii)*), the adopted sequence of actions helped to lower the number of failures in the relevant phase. On the other hand, a number of falls during the release phase have been registered, in particular for objects with a challenging combination of relatively high weight and radius, that are difficult to be held with the thumb and index only. Nevertheless, the idea of loosening the grasp to let the bottle set upright by itself is regarded as a favorable solution for its simplicity and effectiveness.

In order to prove the validity of the proposed method, the accuracy results obtained in this work are compared with other works addressing grasp learning and mobile manipulation. To guarantee the comparability of the results, amongst the articles presented in Related Work, only the ones carrying out a validation procedure similar to the one described in Validation are considered. In this view, the overall 88% accuracy could be regarded as a satisfactory result, as in^13^ Wang and colleagues obtained an overall 80% accuracy in a vision-based mobile manipulation task. The result is referred to evaluation trials performed in simulation, whereas a reportedly degraded performance when moving to the real robot is observed. In addition, Osa and co-workers^16^ obtained a grasp accuracy of the 90% on a set of rigid objects. To cope with a single deformable object (a plastic bag full of cables), the model underwent a further fine-tuning session to reach an 80% accuracy, starting from an initial 40%. For the sake of completeness, it must be pointed out that methods outperforming the presented one are available. In the approach presented in^14^ by Kalashnikov and collaborators, a robotic arm is trained to successfully grasp an object from a cluttered bin in up to the 96% of the cases. Nevertheless, it is important to notice that the learning leans on a very large number of samples (580,000) collected by several robots working in parallel, a setup difficult to apply in a service robotic setting.

## Conclusions

An approach for the optimization of a task-oriented grasping strategy based on Bayesian optimization with transfer learning capabilities is presented. A PAL Robotics TIAGo mobile manipulator has been used as a development platform. The values of the 3 actuated joints of the Hey-5 hand are optimized, together with the height at which the end-effector is positioned to perform the grasp. A global model is obtained leveraging the information acquired during the training of 3 object-specific models on as many plastic bottles. Each individual model is obtained with a two-stage optimization procedure: after 50 iterations, the training is stopped and the domain of the exploration is limited to the most promising sets of parameters. In the 25 iterations of the second (and last) phase of the training, a consistent increase of the successful tasks is observed, demonstrating the effectiveness of the proposed optimization strategy. The global model is employed to predict the optimal grasping strategy for a set of new objects, demonstrating the capability to generalize to previously unseen items. The BO algorithm demonstrates to be efficient for the tuning of the grasping task, *i.e.*, guaranteeing adequate performance (achieving a success rate of the 88%) in a limited number of iterations (80 trials to train a grasping from scratch).

Future work should strive to increase the dimensionality of the problem, possibly until the 6 DoF defining the end-effector pose are encompassed in the optimized parameters. In addition, the bottle releasing strategy will be optimized in order to maximize its performance.

## Data Availability

The datasets generated during and/or analysed during the current study are available from the corresponding author on reasonable request.
